# The Effects of 5-Fluorouracil/Leucovorin Chemotherapy on Cognitive Function in Male Mice

**DOI:** 10.3389/fmolb.2021.762116

**Published:** 2021-10-28

**Authors:** Thomas Groves, Christa Corley, Stephanie D. Byrum, Antiño R. Allen

**Affiliations:** ^1^ Division of Radiation Health, University of Arkansas for Medical Sciences, Little Rock, AR, United States; ^2^ Department of Pharmaceutical Sciences, University of Arkansas for Medical Sciences, Little Rock, AR, United States; ^3^ Neurobiology and Developmental Sciences, University of Arkansas for Medical Sciences, Little Rock, AR, United States; ^4^ Department of Biochemistry and Molecular Biology, University of Arkansas for Medical Sciences, Little Rock, AR, United States; ^5^ Arkansas Children’s Research Institute, Little Rock, AR, United States

**Keywords:** 5-fluorouracil, hippocampus, neurovascular, cognition, mice

## Abstract

5-Fluorouracil (5-Fu) and leucovorin (LV) are often given in combination to treat colorectal cancer. 5-Fu/LV prevents cell proliferation by inhibiting thymidylate synthase, which catalyzes the conversion of deoxyuridine monophosphate to deoxythymidine monophosphate. While 5-Fu has been shown to cause cognitive impairment, the synergistic effect of 5-Fu with LV has not been fully explored. The present investigation was designed to assess how the combination of 5-Fu and LV affect cognition in a murine model. Six-month-old male mice were used in this study; 15 mice received saline injections and 15 mice received 5-Fu/LV injections. One month after treatment, the elevated plus maze, Y-maze, and Morris water maze behavioral tasks were performed. Brains were then extracted, cryosectioned, and stained for CD68 to assay microglial activation and with tomato lectin to assay the vasculature. All animals were able to locate the visible and hidden platform locations in the water maze. However, a significant impairment in spatial memory retention was observed in the probe trial after the first day of hidden-platform training (first probe trial) in animals that received 5-Fu/LV, but these animals showed spatial memory retention by day 5. There were no significant increases in inflammation as measured by CD68, but 5-Fu/LV treatment did modulate blood vessel morphology. Tandem mass tag proteomics analysis identified 6,049 proteins, 7 of which were differentially expressed with a *p*-value of <0.05 and a fold change of >1.5. The present data demonstrate that 5-Fu/LV increases anxiety and significantly impairs spatial memory retention.

## Introduction

The development and improvement of chemotherapeutic treatments has led to a higher survival rate for cancer patients. However, cancer survivors that undergo chemotherapeutic treatment often have long-term cognitive impairments referred to as “chemobrain,” or “chemofog.” This cognitive dysfunction is characterized by attention and memory problems, lack of concentration, and difficulties with multi-tasking following chemotherapeutic treatment (Matsuda et al., 2005; Hermelink et al., 2007; Vardy and Tannock, 2007; Hutchinson et al., 2012; Wefel and Schagen, 2012). These symptoms are often only temporary but have been reported to persist for months-to-years in patients already in cancer remission (Schagen et al., 1999; Wefel et al., 2010; Koppelmans et al., 2012).

Even though there is an abundance of literature on chemobrain, repeated studies have shown that cognitive impairments seen in patients have unclear clinical significance. For example, most studies examining cognitive dysfunction following chemotherapeutic treatment have had patients younger than 65 years old, even though cancer occurs most-often in patients older than 65-years old (Schagen et al., 1999; Brezden et al., 2000). One study in cancer out-patients older than 65 years old found that the chemotherapy group showed no impairment from baseline 6 months post-treatment (Minisini et al., 2008). Another study found subjective cognitive deficits in breast cancer patients with a mean age of 70 years preceding and following treatment (Hurria et al., 2006). Other factors, such as anxiety, depression, stress, and inflammation may also influence the cancer-related cognitive dysfunction seen in patients (Ahles and Saykin, 2007; Joly et al., 2011). The current study utilized aged male mice which are particularly useful for evaluating the impact of chemotherapeutic agents on cognitive functions.

5-Fu is an antimetabolite that only differs from uracil because of a fluorine atom at the C-5 position of the pyrimidine base (Heidelberger et al., 1957; Schilsky, 1998). 5-Fu is incorporated into tissues through the RNA synthetic pathway: 5-Fu is first converted into fluorouridine (FUrd) *via* uridine phosphorylase and then into deoxyuridine monophosphate (dUMP) by uridine kinase (UK) (Miura et al., 2012) Working to prevent cell proliferation, 5-Fu primarily inhibits the enzyme thymidylate synthase, thus blocking the thymidine formation required for DNA synthesis (Hartmann and Heidelberger, 1961; Pinedo and Peters, 1988; Parker and Cheng, 1990; Chu et al., 2003; Longley et al., 2003). Thymidine nucleotide biosynthesis differs from other deoxyribonucleotides in that it first appears within cells as deoxythymidine monophosphate (dTMP), while other deoxyribonucleotides of DNA are created at the diphosphate level. Therefore, ribose-containing thymidine mononucleotides are not just normally found within cells. dTMP is formed *via* a reaction that alters the pyrimidine base of dUMP: Thymidylate synthase catalyzes the methylation of the 5-position of dUMP with the donor cofactor 5,10-methylenetetrahydrofolate (5,10-methylene-THF) (Hartmann and Heidelberger, 1961). Thymidylate synthase is an important target for cancer chemotherapy because it provides the only intracellular *de novo* source of thymidylate, one of four essential nucleotides for the biosynthesis of DNA.

One method of increasing the reduced folate pool in both experimental and clinical settings has been through the use of Leucovorin (LV), a 5-formyl derivative of tetrahydrofolate that is metabolized into 5,10-methylene-THF in the folate pathway. Several studies have shown that co-administration of 5-Fu and LV in patients with different types of cancer lead to increased 5,10-methylene-THF and TS inhibition. For example, when this combination therapy was given in patients with colorectal cancer, 5,10-methylene-THF levels increased several fold, while TS inhibition was maintained at 75% after 48 h, compared to just 50% inhibition in the group of patients that were only administered 5-Fu.

During the mid-1990s, adjuvant treatment of 5-Fu/LV was firmly established as beneficial for patients with stage III colorectal cancer. The National Surgical Adjuvant Breast and Bowel Project found that for both overall survival and disease-free survival, 5-Fu/LV was superior to the previous standard adjuvant treatment (5-Fu and levamisole) recommended by the national institute of health (NIH) for stage III CRC (Wolmark et al., 1999). Another important phase III trial conducted by Intergroup 0089 assessed the efficacy of 5-FU/LV based on the two most common administration regimens: Mayo Clinic (5-Fu and low-dose LV) and Roswell Park (5-Fu and high-dose LV). Both dose schedules demonstrated similar efficacy for 5 years overall survival and disease-free survival, showing that patients would be able to choose the treatment regimen that would maximize their survival outcome (Haller et al., 2005). Finally, a meta-analysis by Sargent et al., (2001) of seven phase III trials revealed that adjuvant chemotherapy with 5-FU/LV improved overall survival and time to tumor recurrence for patients that received treatment after surgery compared to those that only had surgery (Sargent et al., 2001).

Microglia have been increasingly implicated in neurovascular development, maintenance, and complexity, as they have been observed to be in close proximity to the neurovasculature (Checchin et al., 2006; Rymo et al., 2011; Zhao, 2018). They are also capable of releasing vascular endothelial growth factor, which is an important regulator in the development and organization of blood vessels (Thomas, 1996). In a study by Fonseca et al., sustained microglial activation was accompanied with reduced neural stem cell proliferation in the subventricular zon and microglial remodeling. Microglial uniformity decreased and microglia previously associated with the vasculature had retracted processes and were located further away from the niche. Changes in microglial quantity also affect the neurovasculature, as depleted retinal microglia has been shown to reduce vascular density (Checchin et al., 2006; Solano Fonseca et al., 2016). Vascular density is reduced in the aging subventricular zon, which is likely an important contributor to neural stem cell dysfunction (Checchin et al., 2006; Solano Fonseca et al., 2016). Despite the continued interest in the central nervous system vasculature, there is still a need to determine how microglia influence vascular remodeling in adult neurogenic niches. Furthermore, more research is required to understand how certain pathologies affect microglial functions in relation to blood vessel development and maintenance.

Several behavioral studies that have examined the cognitive effects of 5-Fu treatment by itself, there have been few studies that have tested the combination of 5-Fu and LV. In studies conducted by Mustafa et al., (2008) and El Beltagy et al., (2010), Lister-hooded rats were given 5 and 6 I.V. injections, respectively, of 5-Fu (20 mg/kg) and LV (20 mg/kg) every other day for 2 weeks and performed the novel location recognition task. For both studies, rats that received the 5-Fu and LV combination failed to discriminate between the two object locations and spent more time on the object in the familiar location. These studies suggest that 5-Fu and LV induced a spatial memory deficit in rats (Mustafa et al., 2008; ElBeltagy et al., 2010). While these studies are promising, there is still a need to determine how this drug combinations affects other aspects of memory and brain function (short-term memory, long-term memory, locomotion, etc.). Even with several clinical studies describing long-term cognitive deficits and CNS damage following systemic chemotherapeutic exposure, the underlying mechanism for these effects are not fully understood. In addition, the roles that inflammation and the neurovasculature play are completely unknown. Thus, the hypothesis being tested is that 5-Fu/LV combination therapy will result in increased activated microglia, alterations in blood vessels within the dorsal DG, and cognitive impairment.

## Materials and Methods

### Animals and Study Design

Six-month-old male C57Bl6/J wild-type mice (*n* = 30) purchased from the Jackson Laboratory (Bar Harbor, ME) were used for the experiments. The mice were housed and aged under a constant 12 h light: 12 h dark cycle. Food and water were provided ad libitum. All procedures were approved by the Institutional Animal Care and Use Committee at UAMS.

### Injection Paradigm

**SCHEME 1:**
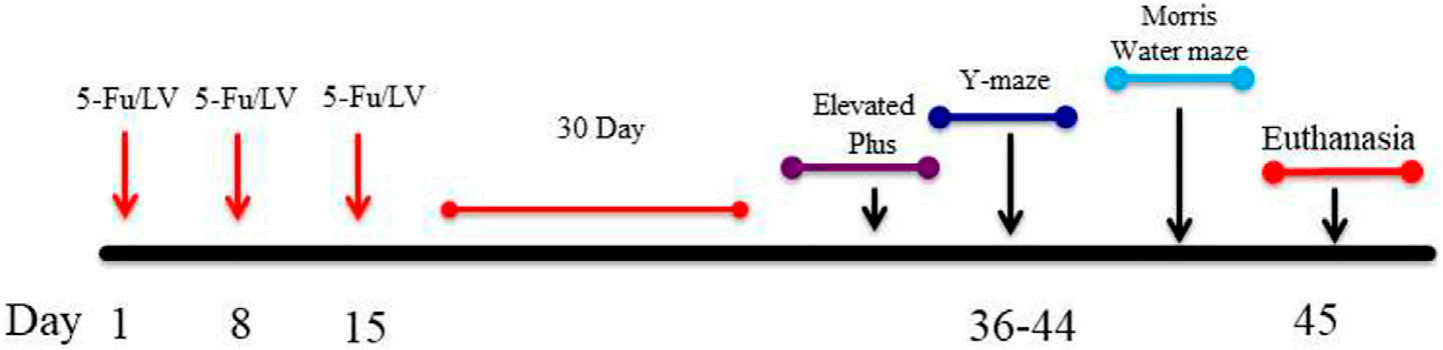
Showing experimental design. Six-month-old C57BL/6J male mice received intraperitoneal injections weekly for 3 weeks of either saline (0.9% sodium chloride) or 5FU/LV. 30 days after last injections, behavioral testing was initiated.

In order to determine if 5-Fu/LV affects cognition, our study consists of two groups of 6-month old C57Bl6/J wild type male mice: 1) a control group that receives only 0.09% Saline (*n* = 15) intraperitoneal injections, and 2) a group that was co-administered 5-Fu (50 mg/kg) + LV (90 mg/kg) (*n* = 15) over a 3-week period on days 1, 8, and 15 (see Scheme 1). Each intraperitoneal injection consists of 340 µL of solution. The dosages of 5-Fu and LV translate to human equivalent doses that are below clinical dose. Drugs were diluted with sterile saline and stored per the manufacturer’s instructions each day between 0900 and 1,200 h. Drugs were mixed immediately prior to injections. We euthanized the animals and extracted the brains 30 days after the final injection.

### Elevated-Plus Maze

The EPM was used to test for anxiety-like behavior and was conducted when mice were 35 weeks old. The apparatus was made of blue acrylic plastic with two sets of opposing arms (open arms: 50 × 10 cm; closed arms: 50 × 10 × 40 cm), 50 cm from the ground. Mice (*n* = 30) were placed individually in the center of the maze and allowed to start exploring the maze freely for 5 min. The maze was cleaned between mice using 20% ethanol. Time spent and the number of entries into each arm was recorded with Ethovision XT software 11 (Noldus Information Technology, Sterling, VA).

### Y-Maze

Short-term spatial memory was assessed with the Y-maze task for mice at 35 weeks old. The Y-maze apparatus was composed of three arms (45 × 15 × 30 cm), each designated as the start, familiar, and novel arm. Each arm contained a different visual cue. The Y-maze task had two 5-min trials that were performed 4 h apart; the training trial and the retention trial. For the training trial, mice (*n* = 30) were placed in the start arm and were allowed to explore only two arms (start and familiar), with the novel arm being blocked. For the retention trial, mice had access to explore all three arms. Allocation of arms (start, familiar, and novel) was counterbalanced within each experimental group. Mice were tracked and videos were recorded with Ethovision XT.

### MWM

Hippocampus-dependent cognitive performance was tested with the Morris water maze task. A circular pool was filled with opaque water maintained at 24°C and spatial clues were placed around it. The Morris water maze task is composed of three phases: 1) Visible platform learning, 2) Hidden platform learning, 3) Probe trials. For visible platform learning, animals were trained to locate a platform that was slightly above the water and that had an object placed on it. Hidden platform learning involved training mice to locate the platform below the water on days 3–5. Probe trials, conducted on days 3–5 following hidden platform training, involved removing the platform from the pool and allowing mice to explore the arena for 60 s.

Mice (*n* = 30) were placed at the edge of the pool in one of nine randomized locations, with the start location and platform location dependent on the phase. Swimming paths were video-recorded using the EthoVision XT video tracking system, set at 6 samples/second. Two sessions consisting of three trials each were conducted 2 h apart every day. A trial ended when the mouse located the platform and remained on it for three cumulative seconds. If the mouse did not locate the platform after 60 s, it was guided to it by the examiner, where it remained for 10 s. Probe trials were performed 1 h after the last trial of hidden platform learning.

### Behavior Analysis

Data were expressed as means ± the standard error of the mean (SEM). All behavioral statistical analyses were conducted with Prism 6.0 software (GraphPad). Elevated-plus and Y-maze were analyzed by a one-way analysis of variance (ANOVA). Visible- and hidden-platform water maze learning curves were analyzed by two-way repeated measures ANOVA. Separate analyses were conducted for the visible- and hidden-platform learning curves. For analysis of performance in the probe trials, we used one-way ANOVAs. Holm’s post-hoc tests were performed on all behavior data. Differences were considered to be statistically significant when *p* < 0.05.

### Tissue Processing Lectin and CD68

Mice were first injected in the posterior vena cava with 150 μL Dylight 488 Tomato Lectin (Vector Laboratories, DL-1174). Next, animals were transcardially perfused with 25 ml 0.9% saline solution containing 0.05 ml heparin, followed by 25 ml 4% paraformaldehyde. Both the tomato lectin injection and the transcardial perfusion were performed under anesthesia. Brains were removed, post-fixed overnight 4°C, and cryoprotected in 20% sucrose in 0.1 M phosphate buffer at 4°C. Brains were then cryosectioned at 40 μm thickness. Every fifth tarting from where the hippocampus was first visible, for a total of 5 sections per mouse (*n* = 4), was used for immunohistochemical detection of the neurovasculature. Mice were sacrificed at 38 weeks of age.

Sections were incubated with rat anti-mouse CD68 (Abcam, ab53444) at 1:1,000 concentration in 0.1 M PBS with TSABB-3% NRS overnight at 4°C. Sections were then rinsed 3 times for 30 min at room temperature and subsequently incubated for 2 h with a biotinylated anti-rat IgG (Vector Laboratories, BA-4001) in TSABB-3% NRS at room temperature. Three rinses were performed for 30 min at room temperature and then sections were incubated in fluorescein cyanine 3 (PerkinElmer, SAT704A001EA) at 1:75 concentration in amplification diluent for 30 min at room temperature. Sections were rinsed 3 times for 30 min at room temperature, placed on slides, and then coverslipped in Vectashield mounting medium with DAPI (Vector Laboratories, H-1500).

Images were acquired using a Zeiss Axioimager microscope under non-saturating exposure conditions. CD68-positive cells were quantified using the Stereoinvestigator optical fractionater workflow. Contours for the dentate gyrus (DG) and cornus ammonis (CA) areas 1–3 were traced on the right hemisphere for every section. The counting frame and SRS grid layout were defined as 75 × 75, and CD68-positive cells were counted at 20 × magnification. Cell population (N) was calculated as N = $\sum Q\left(\frac{t}{h}\right)\left(\frac{1}{asf}\right)\left(\frac{1}{ssf}\right)$ . The volume of the region of interest was calculated using the Cavalieri method, and cell density was calculated as N/Estimated volume (µm³) for both the DG and CA1-3 (Gundersen and Jensen, 1987).

To trace and quantify blood vessels, contours for the dorsal DG were traced on the right hemisphere of the first 2 sections of each slide. Next, the acquisition SRS image stack workflow in Stereoinvestigator (Ver. 11, Microbrightfield, Inc., Williston, VT) was used to take image stacks at a depth 40 μm. Images were then uploaded into Neurolucida (Ver. 11, Microbrightfield, Inc., Williston, VT), where the interactive AutoNeuron (version 6.02) function was used to trace blood vessels. These files were then uploaded into Neurolucida Explorer (Ver. 11, Microbrightfield, Inc., Williston, VT) for analysis of the length, volume, and diameter of blood vessels. The data obtained by Neurolucida Explorer analysis were compared by a *t*-test. Post hoc pair comparisons were carried out using the Holm-Sidak test. Values of *p* < 0.05 were considered to be statistically significant.

### Protein Isolation

The hippocampus was removed and placed in 400 µL of RIPA lysis buffer (10 mM Tris-Cl pH 8.0, 1 mM EDTA, 0.5 M EGTA, 1% Triton X-100, 0.1% sodium deoxycholate, 0.1% SDS, 140 mM NaCl). The tissue was homogenized on ice, incubated for 30 min on ice, and then centrifuged at 20,000 × *g* for 10 min at 4°C. The supernatant was transferred to a new microcentrifuge tube and stored at −80°C until processing. The Compat-Able™ Protein Assay Preparation Reagent Kit (Thermo Scientific™) was used to eliminate EGTA as an interfering substance for the BCA Pierce TM BCA Protein Assay Kit (Thermo Scientific™).

### Tandem Mass Tag Proteomics Analysis

Tandem mass tag technology is a powerful tool for precise and accurate quantitative proteomics. This method has been widely used to characterize protein expression profiles and investigate and compare functional changes at the protein level. The protocol involves extraction of proteins from cells or tissues followed by reduction, alkylation, and digestion. Samples from each biological condition were labeled with 1 of the 6 isobaric tags of the Tandem mass tag reagent. Resulting peptides were pooled at equal concentrations before fractionation and data acquisition. The Tandem mass tag-labeled samples were analyzed by LC–MS/MS. In an MS1 scan, same-sequence peptides from the different samples appear as a single unresolved additive precursor ion. Fragmentation of the precursor ion during MS/MS (MS2) yields sequence-informative b- and y-ions, and further fragmentation by MS3 (SPS) provides quantitative information as distinct masses between m/z 126 and 131 representing the “different” reporter ions. The reporter ion intensity indicates the relative amount of peptide in the mixture that was labeled with the corresponding reagent.

### Bioinformatics Analysis

Ingenuity Pathway Analysis was used to investigate affected signaling pathways based on the deregulated protein. The ROAST method was performed to investigate unidirectional and bidirectional regulation of significant proteins.

## Results

### Elevated Plus Maze

**FIGURE 1:**
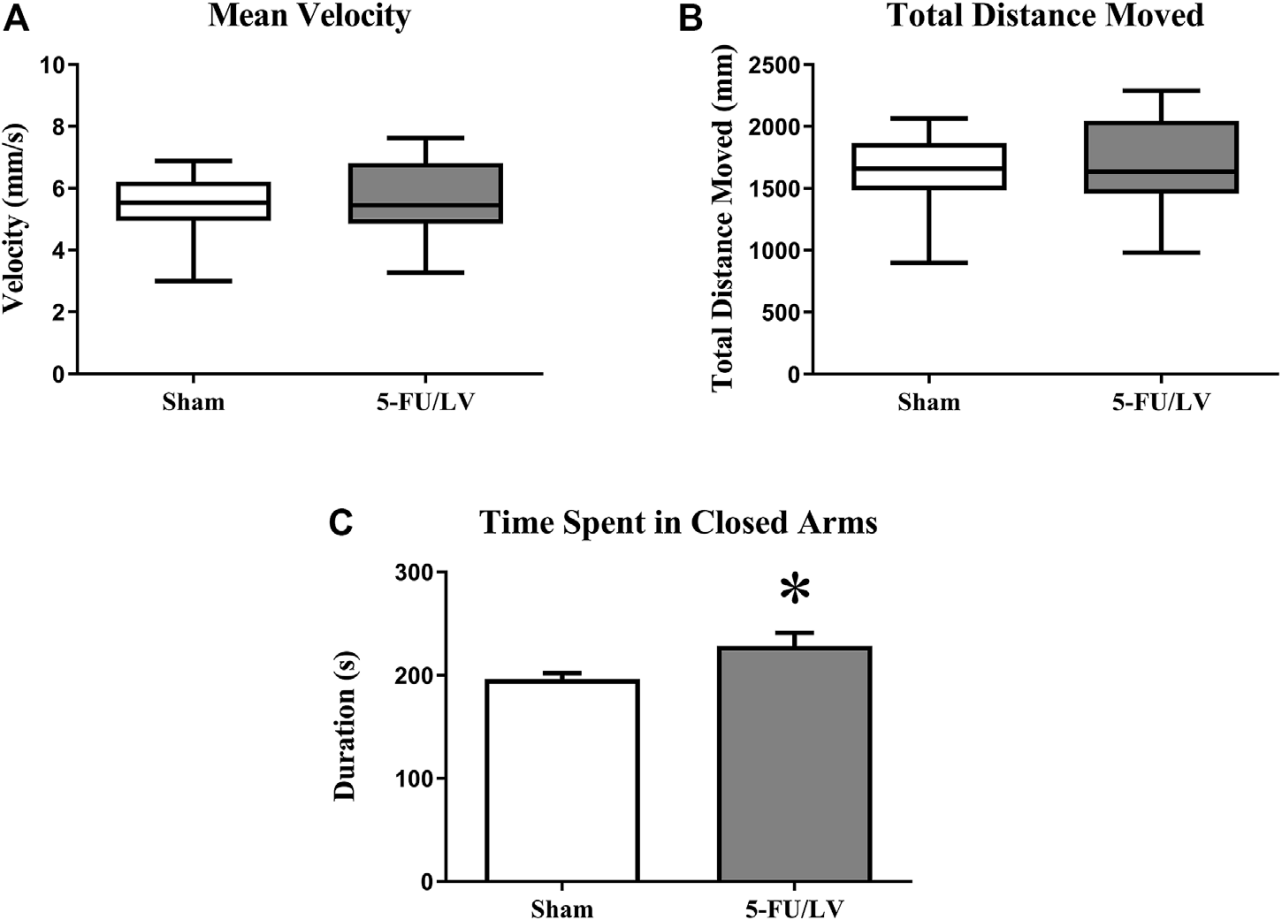
Anxiety-related behavior as determined in the elevated-plus maze. 5Fu/LV treated mice had significantly more closed arm entries compared to sham mice. Average ± SEM (*n* = 15 per treatment Group) *p < 0.05.

To examine anxiety-like behavior in mice treated with 5-Fu/LV, the elevated plus maze task was performed 4 weeks after chemotherapy administration. The elevated plus maze task showed that there was no significant difference in velocity (*t* = 0.37, *p* = 0.355) or distance moved (*t* = 0.38, *p* = 0.360) between mice treated with saline versus 5-Fu/LV. However, there was a significant difference in entries into the closed arm between treatment groups (Figure 1; *t* = 2.18, *p* < 0.05). This indicates that 5-Fu/LV treatment significantly increased anxiety behavior.

### Y-Maze

**FIGURE 2:**
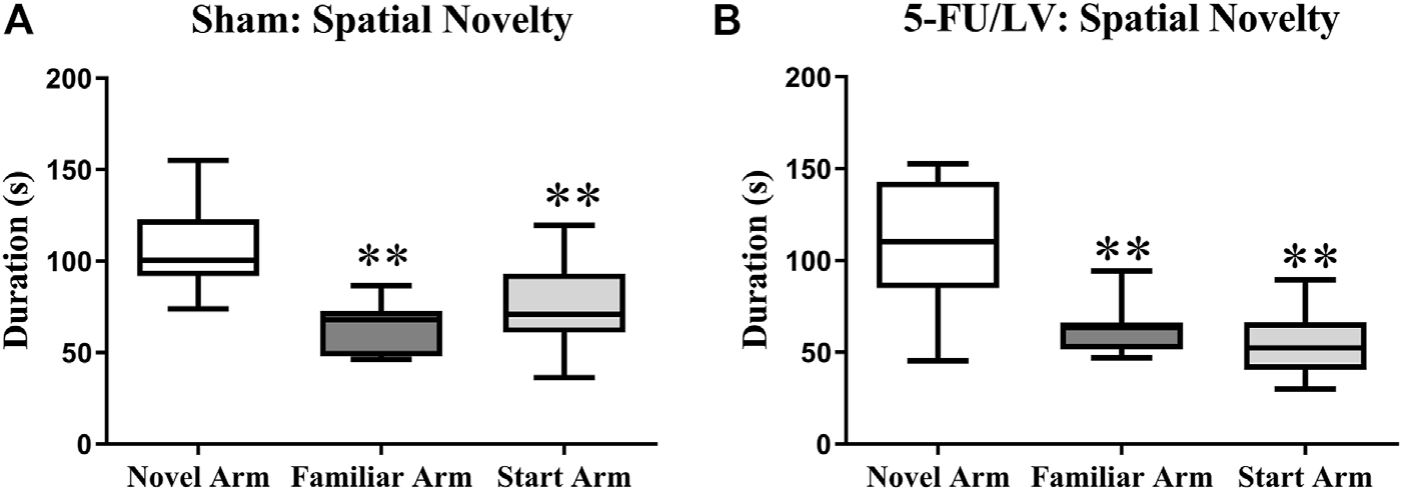
Duration of time spent in the novel arm for the Y-maze task. **(A,B)** Both treatment groups spent a significant duration of time in the novel arm compared to both the familiar arm and the start arm. Average ± SEM (*n* = 15 per treatment Group) *p < 0.05.

The Y-maze task was performed 4 weeks after chemotherapy administration to examine short-term memory function. Both the saline-treated (F_(2, 21)_ = 8.47, *p* < 0.01; Figure 2A) and 5-Fu/LV–treated (F_(2, 21)_ = 9.60, *p* < 0.001; Figure 2B) mice spent significantly more time exploring the novel arm than the familiar and start arms. This suggests that short-term memory was preserved for all treatment groups.

### Morris Water Maze

**FIGURE 3:**
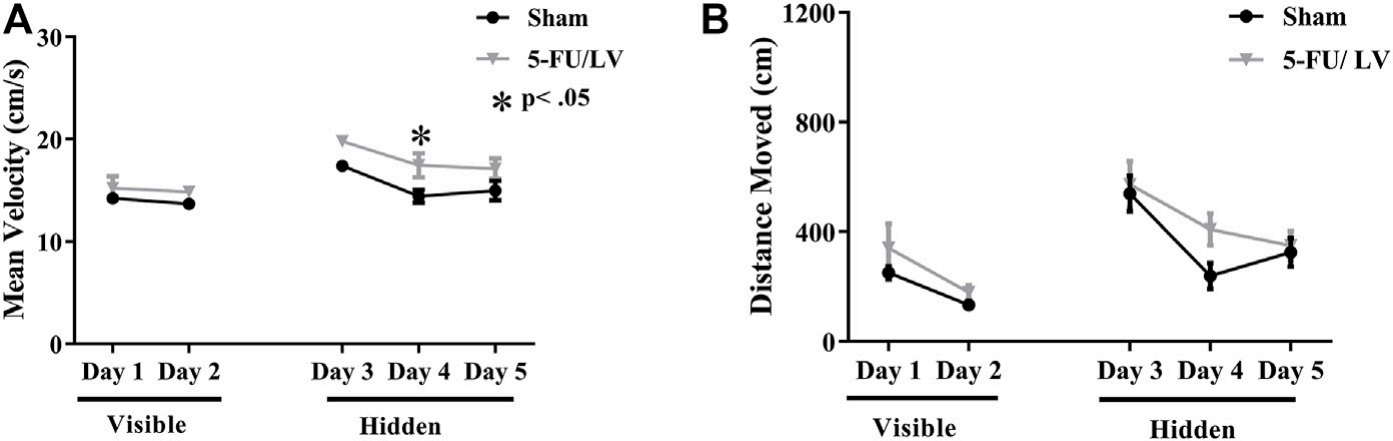
Learning curve data for the MWM task. **(A,B)** 5-Fu/LV treated mice had a significantly higher average velocity on day 4 only compared to sham mice. Average ± SEM (*n* = 15 per treatment Group) *p < 0.05, **p < 0.01.

Spatial memory was assessed with the Morris water maze. With respect to velocity (Figure 3A), there were not significant differences in treatment-by-day interactions (F_(4, 56)_ = 0.75, *p* = 0.56), but there was a significant effect of treatment (F_(1, 14)_ = 7.86, *p* < 0.05) and time (F_(4, 56)_ = 11.89, *p* < 0.001) on velocity. Post hoc analysis revealed a significant difference on day 4 (*p* < 0.05; Figure 3A) resulting from treatment. A decrease in path length (i.e., distance moved) to the platform represents improvement in spatial learning and memory. Repeated-measures ANOVA of distance traveled revealed no significant differences in treatment-by-day interactions (F_(4, 56)_ = 0.57, *p* = 0.68), but there was a significant effect of time (F_(4, 104)_ = 22.61, *p* < 0.001). Post hoc analysis did not revealed any significant differences resulting from treatment.

**FIGURE 4:**
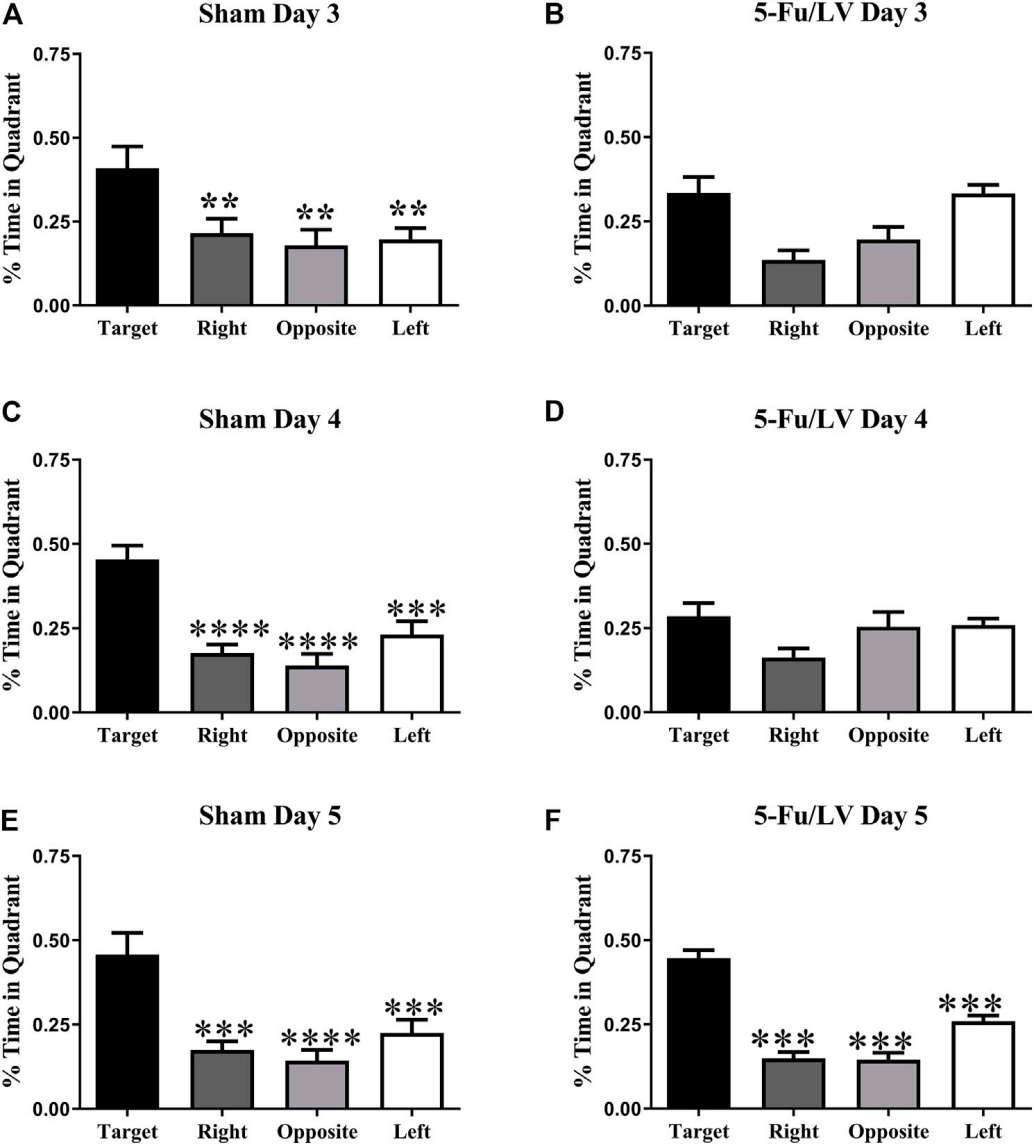
Percent of total time spend in each quadrant for the MWM task probe trial on day 3. Sham mice spent significantly more time in the target quadrant compared to the other three quadrants [Fig. **(A)**: Day 3], [Fig. **(C)**: Day 4], [Fig. **(E)**: Day 5]. 5-Fu/LV treated mice spent their time randomly searching quadrant [Fig. **(B)**: Day 3], [Fig. **(D)**: Day 4]. However, 5-Fu/LV spent significantly more time in the target quadrant day 5 [Fig. **(F)**]. Average ± SEM (*n* = 15 per treatment Group) *p < 0.05, **p < 0.01, ***p < 0.001, ****p < 0.001.

A probe test in which the platform was removed from the water maze was performed on days 3–5. The percent of time spent in each quadrant was then used to determine spatial memory retention. As shown in Figure 4A, saline-treated mice spent significantly more time in the target quadrant on days 3 (F_(3, 28)_ = 4.844, *p* < 0.05, Figure 4A), 4 (F_(3, 28)_ = 15.24, *p* < 0.0001, Figure 4C), and 5 (F_(3, 28)_ = 10.9, *p* < 0.0001, Figure 4E). In contrast, 5-Fu/Lv–treated mice day 3 (F_(3, 28)_ = 7.99, *p* < 0.01: Figure 4B), post hoc analysis revealed were not able to distinguish between target and left quadrant. Day 4 5-Fu/Lv–treated mice not spend more time in the target quadrant compared to other quadrants or day 4 (F_(3, 27)_ = 2.50, *p* = 0.08, Figure 4D). During the final probe trial (day 5), the 5-Fu/LV treated mice spent significantly more time in the target quadrant than in the right, opposite, and left quadrants (F_(3, 28)_ = 49.08, *p* < 0.0001, Figure 4F).

### Dorsal Dentate Gyrus Blood Vessels

**FIGURE 5:**
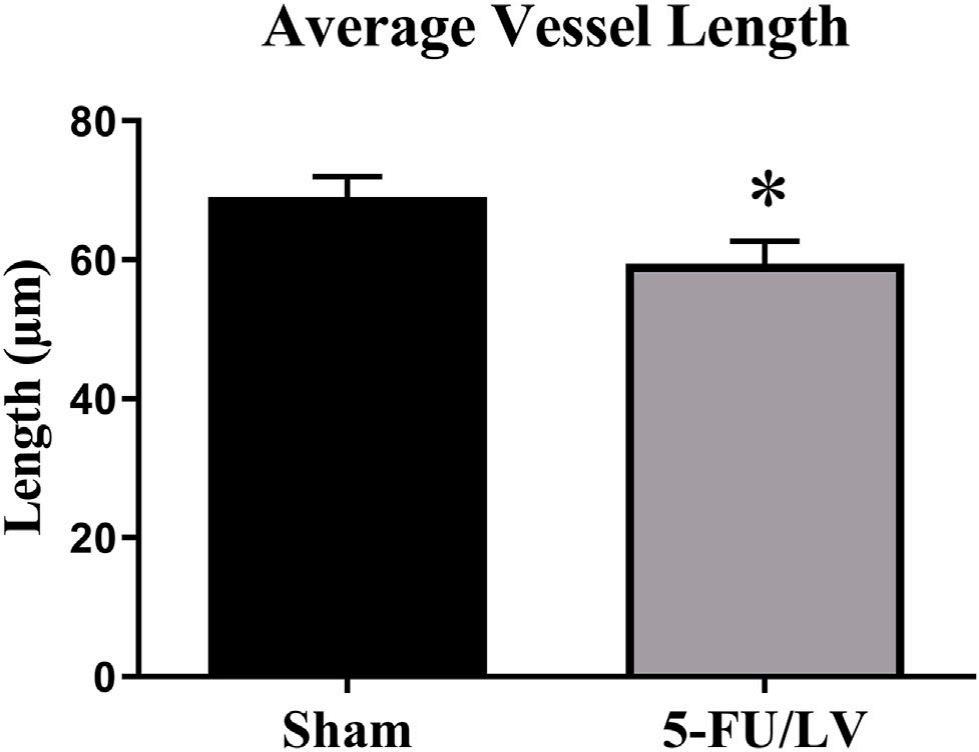
Average blood vessel length in the dorsal DG. 5Fu/LV treated mice had a significant decrease in blood vessel length in the dorsal DG. Average ± SEM (*n* = 5 per group) *p < 0.05.

Tomato lectin immunostaining was performed to determine if 5-Fu/LV treatment led to alterations in the neurovasculature that could account for cognitive dysfunction. To estimate blood vessel volume, length, and diameter in the dentate gyrus, vessels were traced with Neurolucida and quantified with Neurolucida Explorer. Confocal laser microscopy images of double-labeled immunofluorescence for DAPI (in blue) and tomato lectin (in green) were captured. The analysis of tomato lectin-labeled vessels revealed no significant changes in average blood vessel volume in the dorsal dentate gyrus (*t* = 0.006, *p* = 0.49) between saline- and 5-Fu/LV–treated groups. Likewise, no significant difference was found between groups with respect to the average blood vessel diameter (*t* = 0.25, *p* = 0.41). However, treatment with 5-Fu/LV induced a significant decrease in blood vessel length (*t* = 0.25, *p* < 0.05; Figure 5).

### Activated Microglia

**FIGURE 6:**
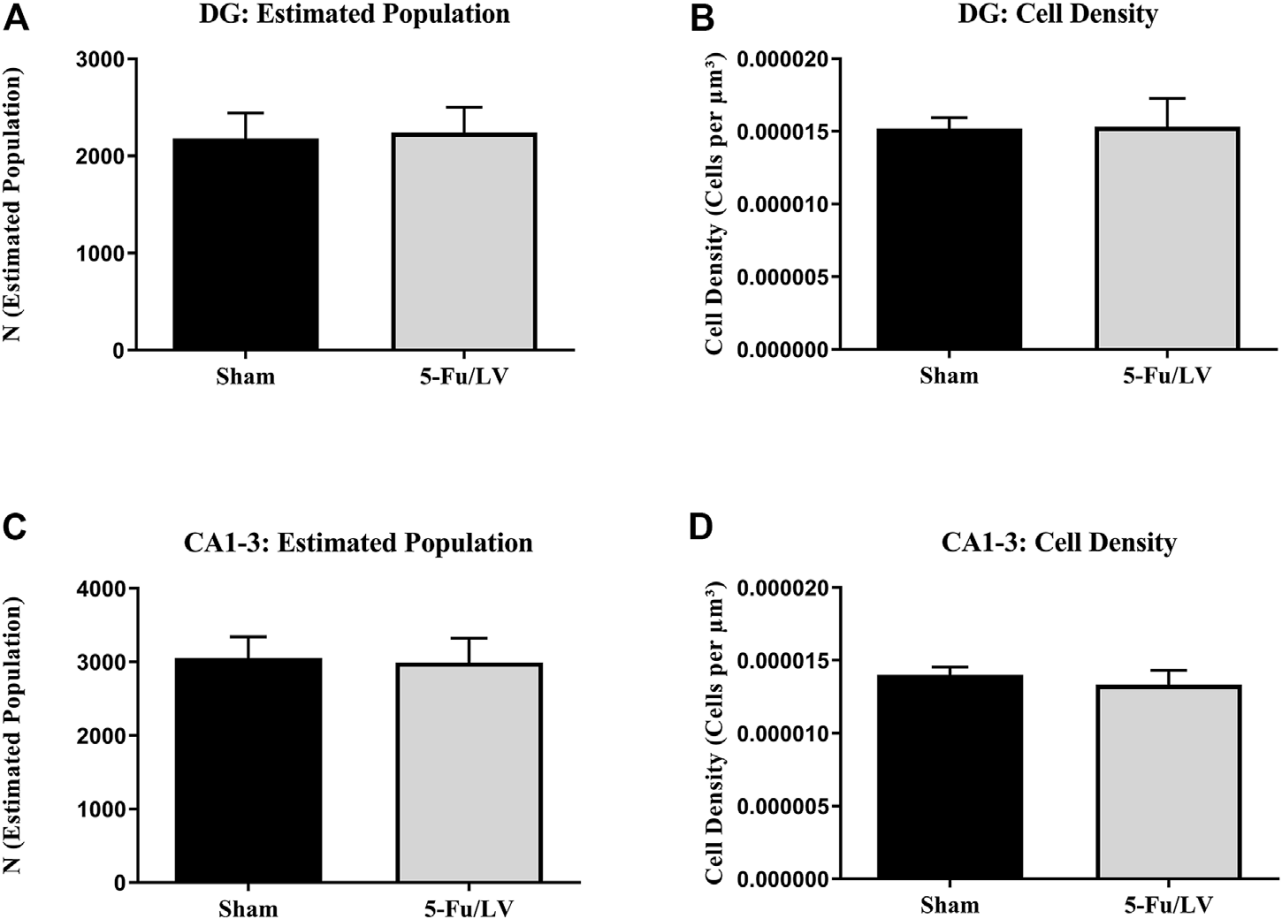
5-Fu/LV treatment does not affect activated microglia activation. The analysis of CD68-labeled cells revealed no significant differences in cell density or estimated cell number in DG [Fig. **(A)** and **(B)**] and CA 1–3 areas [Fig. **(C)** and **(D)**].

CD68 immunostaining was performed to investigate if the activation of microglial cells persisted long-term after chemotherapeutic treatment, as chronic activation of microglia can result in neuronal damage. To estimate microglial activity in the DG and cornus ammonis areas 1–3 (CA1–CA3), cells were counted and quantified using Stereoinvestigator. The analysis of CD68-labeled cells revealed no significant differences in cell density between experimental groups in either the DG (*t* = 0.58, *p* = 0.29; Figure 6A) or CA1–CA3 (*t* = 0.27, *p* = 0.79; Figure 6C). There were also no significant differences in estimated cell number in the DG (*t* = 0.35, *p* = 0.37; Figure 6B) or CA1–CA3 (*t* = 0.34, *p* = 0.37; Figure 6D).

### Proteomics

**FIGURE 7:**
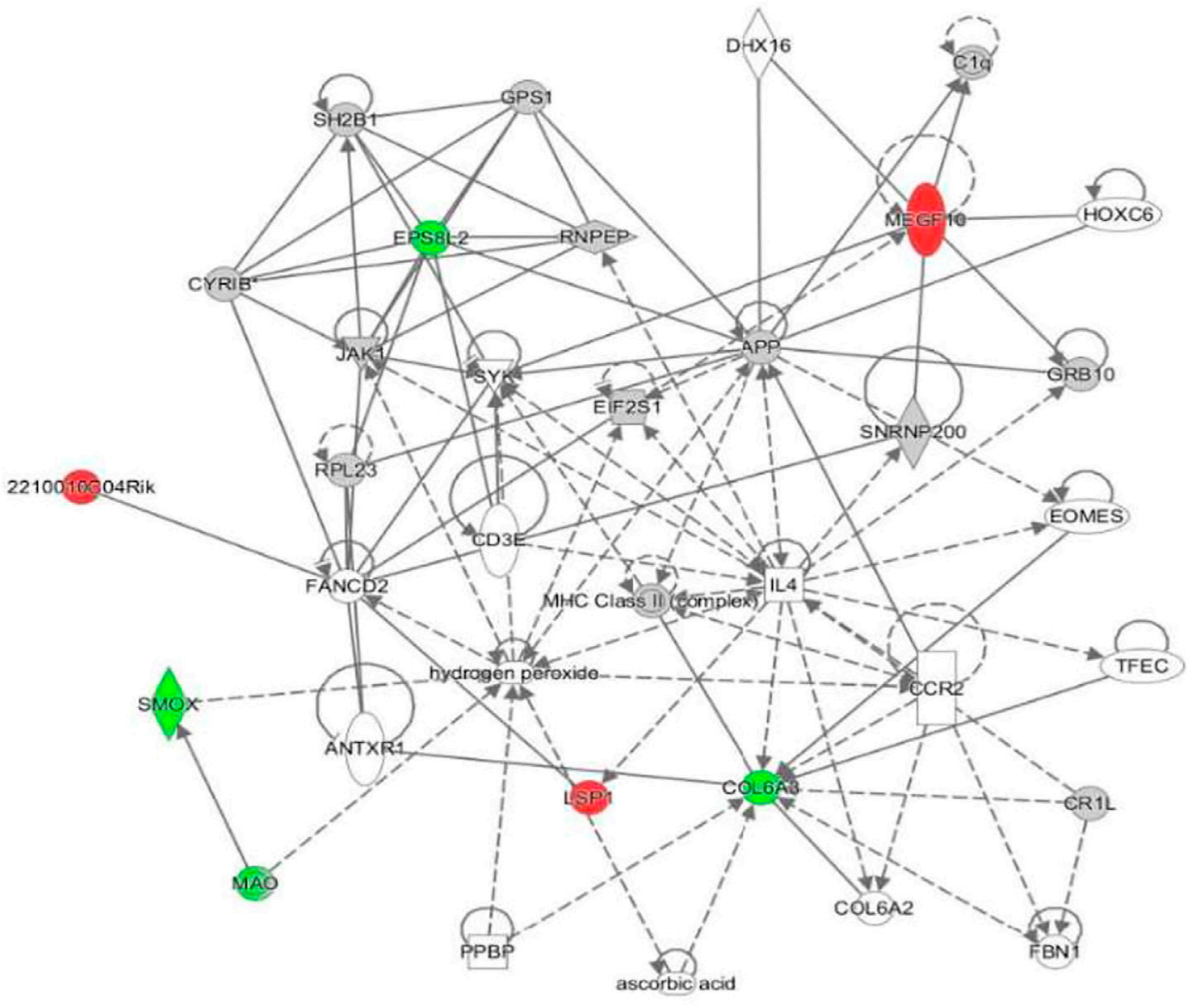
Visual representation of the protein network for hippocampal tissues treated with 5-FU and leucovorin. Nodules represent specific characteristics. Red represents upregulation of proteins. Green represents downregulation of proteins. Gray identifies proteins that were insignificantly expressed but included in the network pathway.

Proteomic analysis was performed to compare proteins expressed between treatment groups of 5-FU/LV treated female mice to saline treated female mice. In this comparison, 6,049 proteins were identified and 7 were differentially expressed with a *p*-value of <0.05 and a fold change >1.5. To achieve a more insightful perspective, datasets for these treatment groups were input to Ingenuity Protein Analysis (IPA) for an output of potential connections or networks to give a perspective on the relationships of protein dysregulations and cognitive dysfunctions. From this analysis, the top dysregulated proteins were identified (Table 1). Also, the top conical pathways include: Spermine and Spermidine Degradation, Melatonin Degradation II, Phenylalanine Degradation IV (Mammalian, *via* Side Chain), Putrescine Degradation III, and Tryptophan Degradation X (Mammalian, *via* Tryptamine) (Table 2). These 5 conical pathways have a common relationship to spermine oxidase (SMOX). In this chemotherapy treated group, spermine oxidase is downregulated. One network was identified in this comparison. (Figure 7). In this network, we found three upregulated proteins: Cordon-bleu WH2 repeat protein like 1 (COBLL1), ELMO domain containing 1 (ELMOD1), and 2210010C04Rik. We found CXXC finger protein 1 (CXXC1) and spermine oxidase (SMOX) were down regulated proteins.

**TABLE 1:** Dysregulated proteins found in 5-FU/LV treatment group versus saline treatment group.

UniProt id	Protein name	logFC	*p* value	Type	Location	Role
Q6DIB5	Multiple epidermal growth factor-like domains protein 10	6.096875	0.004897	Receptor	Plasma membrane	Autophagy and phagocytosis
E9Q9V3	Nuclear receptor corepressor 2	4.050247	0.081694	Coregulator	Nucleus, extracellular matrix	Part of nuclear receptor corepressor complex
Q9D7Y7	Not available	1.913693	8.1E-06	Enzyme	ECM	Proteolysis
Q3UPW5	Spermine oxidase	−4.11619	0.029856	Enzyme	Cytoplasm	Apoptosis and polyamine metabolism
O88493	Not available (Gene: Col6a3)	−4.75009	0.043115	NA	Cell matrix and ECM	Cell adhesion, ECM organization
Q99K30	Epidermal growth factor receptor kinase substrate 8-like protein 2	−5.33513	0.025052	Growth factor	Cytoplasm and plasma membrane	Actin binding, Rac/Rho signaling
O35161	Cadherin EGF LAG seven-pass G-type receptor 1	−6.12814	0.065984	Receptor	Plasma membrane	GPCR, calcium ion binding

**TABLE 2:** Top 5 conical pathways.

Ingenuity canonical pathways	−log (*p*-value)
Spermine and spermidine degradation I	2.97
Melatonin degradation II	2.97
Phenylalanine degradation IV (Mammalian, *via* side chain)	2.43
Putrescine degradation III	2.26
Tryptophan degradation X (Mammalian, *via* tryptamine)	2.18

## Discussion

Anxiety by itself is not maladaptive, as it promotes survival through escape behavior of unnecessary danger. However, excess anxiety can reduce behavioral activity that is crucial for adaptation (Bishop, 2007). Anxiety-like behavior can be assessed in rodents with the elevated-plus maze (EPM), where an animal explores the open and closed arms of a maze raised off of the ground. Increased time in the closed arm is associated with greater anxiety and vice-versa. Here, we show that on anxiety-like behavior was significantly altered in mice when 5-Fu was co-administered with LV.

The Y-maze task is a hippocampal dependent task that is used for measuring spatial recognition memory (Dellu et al., 1992; Conrad et al., 1997; Dellu et al., 2000; Sarnyai et al., 2000; Martin et al., 2003). It does not require rules to be learned and no rewards or punishments are used. The task has two trials; the first trial allows the animal to explore only two arms, where one is blocked, and the second trial allows the animal to explore all three arms. Time spent in each arm, as well as the number of entries, can be used to assess discrimination of novelty. In this study, we show that 5-Fu co-administered with LV did not have any significant effect on spatial recognition memory functioning in mice. These results are consistent with previous studies conducted in mice with chemotherapeutic treatment (Hou et al., 2013; Salas-Ramirez et al., 2015).

The Morris watermaze is used for assessing spatial memory functions, with extensive evidence of it measuring hippocampal-dependent behavior (Morris, 1984; Brandeis et al., 1989; D’Hooge and De Deyn, 2001). Here, we show a cognitive deficit with 5-Fu administration, which is consistent with previous studies (Winocur et al., 2006; Foley et al., 2008; Mustafa et al., 2008; ElBeltagy et al., 2010; Fardell et al., 2012). LV, a vitamin-B derivative that enhances DNA synthesis when metabolized, potentiates the effects of 5-Fu by increasing the conjunction between 5-Fu and the enzyme thymidylate synthetase. Mice treated with 5-Fu/LV showed spatial memory impairment on days 3 and 4 but recovered by the final day of testing. Previous behavioral studies have shown that 5-Fu affects impairs spatial memory functions. In one study, mice that received a combination of methotrexate (37.5 mg/kg) and 5-fluorouracil (75 mg/kg) via intraperitoneal injections for 3 weeks performed the Morris watermaze task and were found to have a spatial memory impairment. In another study, mice were given one I.P. injection of 5-Fu (75 mg/kg) and performed Novel location recognition and Novel object Recognition. Mice showed no short-term preference for either object for both Novel location recognition and Novel object Recognition. While mice did not prefer either object in the long-term trial for Novel object Recognition, there was a preference for the familiar object in Novel object Recognition. This showed that mice failed to discriminate between the two objects. Deficits with object discrimination in NLR were also observed in another study that gave rats 5 intravenous injections of 5-Fu (25 mg/kg) over a 12-day period (Lee et al., 2006; Winocur et al., 2006; Gandal et al., 2008; Mustafa et al., 2008).

Blood vessels serve a variety of roles, including transmission of signaling molecules, recruiting inflammatory cells to the niche, and serving as a scaffold for migrating neural progenitor cells (Palmer et al., 2000; Shen et al., 2004; Aird, 2007; Jones and Wagers, 2008; Riquelme et al., 2008). Degradation of the vascular niche could lead to a reduction of neural progenitor cells available for neurogenesis, which may contribute to aging and neurological diseases (Gopinath and Rando, 2008). The effects of chemotherapeutic drugs on neurovascular remodeling are poorly understood, as differences have been reported in blood vessel density following chemotherapeutic treatment (Cao et al., 2012; Fardell et al., 2012; Lu et al., 2013). In the current study, we did not see a difference in vessel density, but a significant decrease in blood vessel length. Related to blood vessel structure, further research is needed to determine if 5-Fu has an effect on blood flow. A decrease in blood flow may decrease neurogenesis, since the neurovascular endothelium is involved in the transport of nutrients and essential factors to neural precursor cells (Parker and Cheng, 1990). Furthermore, alterations in blood flow may contribute to cognitive impairments, as even slight disruptions can have negative long-term effects. For example, a study by Carlson et al. found changes in cerebral blood flow and persistent cognitive symptoms 12–18 months after chemotherapeutic treatment for breast cancer survivors (Carlson, 2018). Examining how 5-F affects blood flow may help determine the mechanism of 5-Fu-induced cognitive dysfunction.

In order to expand our findings, we explored key protein expression that modulate neuropathies in the hippocampus. We used a TMT approach to evaluate and compare protein expression between the two treatment groups, saline control and 5-FU/Leucovorin. When looking more closely at the network in Figure 1, the upregulated proteins found have a variety of functions. Cordon-bleu WH2 repeat protein like 1 (COBLL1) role in the cell are morphogenesis, interactions, growth, and phosphorylation and it is related to diseases such as organismal death, colorectal carcinomagenesis, colorectal carcinoma, and gastric epithelial cancer amongst others. ELMO domain containing 1 (ELMOD1) is related to function of the ear and is related to hearing loss, and 2210010C04Rik functions relate to apoptosis. The downregulated proteins were CXXC finger protein 1 (CXXC1) and spermine oxidase (SMOX). CXXC1 is involved in double-stranded DNA binding, histone binding, histone lysine N-methyltransferase activity (H3-K4 specific), nucleic acid binding, and protein binding. It is associated with gastrulation failure. SMOX regulates polyamines, spermine, reactive oxygen species, and alpha-methylspermine and play a role in apoptosis.

The proteomics analysis identified spermine oxidase as a key player in the protein regulation. The top dysregulated pathways, which includes Spermine and Spermidine Degradation I and Melatonin Degradation II, are all affected by the polyamine, spermine oxidase. Spermine oxidase was found to be downregulated with a fold change of about −4.15. Interestingly enough, spermine oxidase has roles aging and neurodegenerative diseases. Spermine and Spermidine Degradation I pathway is involved in critical role in regulatory functions in the cells, such as transcriptional, translational, and post-transcriptional processes (Babbar et al., 2007). Polyamines, such as spermine oxidase, have a significant role as an enhancer of antioxidant protection and is also essential in the production of antioxidants needed for normal functionality of the brain (Hussain et al., 2017). As the brain ages, polyamine levels decrease which cause cognitive impairments related to age progression. In Parkinson’s disease, dysregulation in SMOX metabolism can cause discrepancies in dopamine binding (Berezov et al., 2013). In Alzheimer’s disease, excessive stress in the brain, such as oxidative stress, can lead to disruption in polyamine function and could possibly contribute to tau pathologies (Sandusky-Beltran et al., 2019).

Melatonin Degradation II pathway the metabolism of indoleamine melatonin which is a hormone in vertebrate secreted by the pineal gland. It is involved in regulation of circadian and seasonal rhythms (Semak et al., 2005). Melatonin metabolism can be relevant as it effects oxidative stress and inflammation in the brain. Studies have shown that melatonin can reverse ROS activity, elevate Nrf2 levels, and downregulated phospho- CAMP-response element-binding (p-CREB), which is a ligand for phospho-5′AMP-activated protein kinase (p-AMPK) and a regulator of inflammation the hippocampus of traumatic brain injury mouse models (Rehman et al., 2019). Phenylalanine Degradation IV (Mammalian, *via* Side Chain) pathway is involved the conversion of phenylalanine to tyrosine. This process is essential during development for if this process is impaired may lead to neurological impairment (Trepp et al., 2020). Putrescine falls into the family of polyamines along with spermine and spermidine. Like metabolism of spermine and spermidine, putrescine plays a significant role in neurodegenerative disorders and have been found in the cortical and subcortical areas of the brain (Vivó et al., 2001). Putrescine Degradation III pathway essential for managing putrescine levels for normal brain function. Tryptophan Degradation X is necessary for the synthesis and degredation on seratonin in the brain.

Proteomic analysis has become increasing popular to gain a better understanding of disease pathways on the molecular level. Development in mass spectrometry and liquid chromatography has allowed for significant improvement in proteomic analysis with higher resolution, accuracy, and sensitivity on larger scale analysis (Zhang et al., 2013; Wang et al., 2020). Using labeling method such as the TMT or tandem mass tag method, we are able to identify comparative protein levels and post-translational modification in the hippocampus.

From information given from the proteomics, a network was developed by the IPA software that predicts the relationship between molecules in the pathways. Functions associated with this network are cell to cell signaling and interaction, cell function and maintenance, and hematological system development and function Molecules of interest within this network include APP (amyloid beta precursor protein), IL-4, and MHC-Class II complex, CCR2, and hydrogen peroxide, which have a relationship to oxidative stress. Amyloid precursor proteins is an integral protein heavily concentrated in the synapses of the neurons. Its primary function in the brain is unknown, but it is the precursor for the generation of amyloid beta protein, hence its name (Puzzo and Arancio, 2013). IL-4, MHC-Class II complex, and CCR2 play major roles in the immune response during inflammation. An increase in reactive oxygen species (ROS), like hydrogen peroxide, can contribute to several factors such as an accumulation of amyloid beta proteins, inflammation, and microglia activation (Wang et al., 2015). Studies show that hydrogen peroxide increase via the mitochondria mediate cognitive dysfunctions related to Alzheimer’s disease and other age-related dysfunctions (Chen et al., 2014). ROS are the major signaling molecules in the progression of inflammation and related diseases (Mittal et al., 2014). It is controversial whether polyamines increases are beneficial or detrimental to the brain. Although polyamines are essential for brain function, polyamine related protein aggregation may in fact have negative effects on the brain promoting toxic metabolites such as hydrogen peroxide (Berezov et al., 2013).

Chemotherapeutic induced cognitive impairments can be caused by a multi-level cascade of molecular events. Based on the information received from the network and pathways found to be involved in the 5-FU/LV treatment suggest antioxidant therapies to combat oxidative stress. Studies suggest melatonin can be used to attenuate cognitive impairments caused by 5-FU treatments by increasing antioxidant activity by restoring antioxidant-dependent pathways such as Nrf2 (Sirichoat et al., 2020; Suwannakot et al., 2021). Based on the data presented, we can only assume the roles polyamines play in model as this proteomics analysis purely descriptive and further analysis must be applied. With polyamines potential producing hydrogen peroxide, these polyamines could be mediating the molecular dysfunctions in this chemobrain model that are more closely related to age related neurodegeneration.

Under normal physiological conditions, microglia play an important role in regulating synaptic connectivity and pruning within the hippocampus (Schafer et al., 2012). However, persistent neuroinflammation can result in the release of inflammatory cytokines like tumor necrosis factor alpha and interleukin 1-beta from activated microglia, which may cause neuronal and glial damage (Woodcock and Morganti-Kossmann, 2013). This can have a negative effect on neurogenesis and cognition (Ekdahl et al., 2003; Das and Basu, 2008). We found that there was not an increase in activated microglia following chemotherapeutic treatment. This may suggest that 5-Fu/LV administration does not lead to chronic microglial activation. Other studies have demonstrated microglial activation following chemotherapeutic treatment, but differences in experimental design, length of time following chemotherapy administration, and the chemotherapeutic agent used necessitates further studies on the timeframe of neuroinflammation and the role it may play in chemobrain (Seigers et al., 2010; Briones and Woods, 2014). One major caveats to our study is that CD68 does not label infiltrating macrophage that could negatively modulate neurovasculature additional studies investigating expression of IBA1 are warranted.

5-Fu/LV administration resulted in impaired spatial memory function, as determined by the MWM. The mechanism that leads to cognitive deficits following chemotherapeutic treatments is still unknown. We found that there was a change in blood vessel morphology following chemotherapy administration. This alteration in the neurovasculature may contribute to cognitive impairments, as even slight disruptions in cerebral blood flow can have negative long-term effects (Zlokovic, 2008; Zlokovic, 2010). However, the effects of chemotherapeutic drugs on neurovascular remodeling are poorly understood, as differences have been reported in blood vessel density following chemotherapeutic treatment (Seigers et al., 2010; Seigers et al., 2015; Seigers et al., 2016). It is controversial whether polyamines increases are beneficial or detrimental to the brain. Although polyamines are essential for brain function, polyamine related protein aggregation may in fact have negative effects on the brain promoting toxic metabolites such as hydrogen peroxide. Based on the data presented, we can only assume the roles polyamines play in model as this proteomics analysis purely descriptive and further analysis must be applied. With polyamines potential producing hydrogen peroxide, these polyamines could be mediating the molecular dysfunctions in this chemobrain model that are more closely related to age related neurodegeneration. Investigating how 5-Fu/LV chemotherapy affects different aspects of cognition may help elucidate the symptoms seen in patients and help lead to more targeted treatment options.

## Data Availability

The mass spectrometry proteomics data have been deposited to the ProteomeXchange Consortium via the PRIDE [1] partner repository with the dataset identifier PXD028899. Project Name: The Effects of 5-Fluorouracil/Leucovorin Chemotherapy on Cognitive Function in Mice. Project accession: PXD028899.
